# A recombinant antibody toolbox for *Dictyostelium discoideum*

**DOI:** 10.1186/s13104-020-05048-8

**Published:** 2020-04-10

**Authors:** Wanessa C. Lima, Philippe Hammel, Pierre Cosson

**Affiliations:** Geneva Antibody Facility, Faculty of Medicine, University of Geneva, 1 rue Michel Servet, 1211 Geneva, Switzerland

**Keywords:** *Dictyostelium discoideum*, Recombinant antibodies, Sequenced antibodies, Hybridoma, Phage display

## Abstract

**Objective:**

The amoeba *Dictyostelium discoideum* has been a valuable model organism to study numerous facets of eukaryotic cell biology, such as cell motility, cell adhesion, macropinocytosis and phagocytosis, host–pathogen interactions and multicellular development. However, the relative small size of the *Dictyostelium* community hampers the production and distribution of reagents and tools, such as antibodies, by commercial vendors.

**Results:**

For the past 5 years, our laboratory has worked to promote an increased use of recombinant antibodies (rAbs) by academic laboratories. Here we report our efforts to ensure that *Dictyostelium* researchers have access to rAbs. Using hybridoma sequencing and phage display techniques, we generated a panel of recombinant antibodies against *D. discoideum* antigens, providing a useful and reliable set of reagents for labelling and characterization of proteins and subcellular compartments in *D. discoideum*, accessible to the entire *Dictyostelium* community.

## Introduction

For the past seven decades, the social amoeba *Dictyostelium discoideum* has been used as a model organism to elucidate the molecular mechanisms underlying numerous eukaryotic cellular processes, notably multicellular development, cell motility, chemotaxis, macropinocytosis and phagocytosis, endocytic vesicle traffic, cell adhesion, caspase-independent cell death, host–pathogen interactions, and microbial infections [1, 2]. *D. discoideum* is a haploid organism, with a relatively small genome (34 Mb) containing around 12,000 predicted proteins. From an evolutionary perspective, *D. discoideum* diverged from the animal lineage before fungi and yeasts, but after plants. Many cellular functions, absent in plant cells, appeared at this stage, in particular cell motility, chemotaxis, phagocytosis and cell adhesion. Due to the higher rate of evolutionary changes inside the Fungi group, the evolutionary distance between *Dictyostelium* and human is shorter than that between human and yeast [3]. Many gene products lost in fungi are maintained in *D. discoideum*, including orthologs of human genes responsible for a variety of diseases [1, 4].

Despite its relevance as a model organism, the *Dictyostelium* scientific community is relatively small, and this can hamper the development of new tools and techniques. One of the most critical tools in cell biology research are antibodies, extensively used for detection and characterization of proteins and cellular components. The mono- and polyclonal antibodies against *D. discoideum* antigens currently in use have been mostly developed during the 1980s; due to the small size of the community, they are not commercially available [1]. Because mono- and polyclonal antibodies are finite resources, many of these anti-*Dictyostelium* tools are either not widely available or have already been lost forever.

This is a critical situation, and the solution is two-fold. On the one hand, the existing monoclonal antibodies produced by hybridomas need to be secured by determining their aminoacid sequences. On the other hand, the past 15 years have seen an explosion of techniques and tools for discovery of recombinant antibodies (rAbs), notably the phage display technique. These new tools allow the community to enrich the list of rAbs against *D. discoideum* targets. Each time a laboratory engages in the sequencing of a hybridoma or in the discovery and characterization of a new recombinant antibody, it facilitates future work for the whole *Dictyostelium* research community.

For the past 5 years, we have used hybridoma sequencing and phage display technologies to ensure that more and more *Dictyostelium* researchers have access to recombinant antibodies [5]. In this study, we describe a panel of recombinant antibodies against *D. discoideum* antigens, providing a toolbox for detection, characterization and isolation of proteins and subcellular compartments in *D. discoideum*.

## Main text

### Material & methods

#### Hybridoma sequencing

The strategy used for hybridoma sequencing is based on the protocol by Schaefer et al. [6]. Frozen vials of hybridoma cells were thawed, resuspended in pre-warmed PBS, pelleted (2000 rpm, 4 min), and resuspended in 1 ml pre-warmed PBS. 5 × 10^6^ cells were used for RNA extraction according to the manufacturer’s instructions (RNeasy mini kit, Qiagen #74104). cDNA synthesis (1 µg RNA) was done using Superscript™ II reverse transcriptase (ThermoFisher #18064014), and random hexamers (ThermoFisher #SO142).

Following the cDNA synthesis reaction, the heavy (V_H_) and light (V_L_) variable domain sequences were amplified by PCR using degenerate primer sets (500 nM, Integrated DNA Technologies; Additional file 1: Table S1) using GoTaq polymerase (Promega #M7848). PCR conditions were: 95 °C for 90 s; 5 cycles of 95 °C for 30 s, 55 °C for 30 s, 72 °C for 45 s; 20 cycles of 95 °C for 30 s, 63 °C for 30 s, 72 °C for 45 s; 72 °C for 5 min.

The PCR products were column-purified and double-digested with HindIII-Hf and SacII (NEB #R3104 and #R0157). V_L_ products were additionally treated with BfuI and/or BseRI (ThermoFisher #ER1501 and NEB #R0581), to eliminate the myeloma aberrant light chain from the myeloma fusion partner. Digested products were gel-purified (300-400 bp) and cloned into pBSK- vector (GenBank #X52330.1, HindIII-Hf/SacII digested and dephosphorylated). 6–10 clones with the correct fragment size were sequenced to obtain the consensus sequences of the V_H_ and V_L_ domains; definition of the heavy and light chain boundary sequences, based on alignment with germline sequences, was done using the VBASE2 server [7].

#### Antibody conversion into an scFv-Fc format

A recombinant antibody can be made in several formats and flavors; our format of choice is an scFv (single chain Fv) linked to an Fc moiety by a small AAARS linker (Additional file 1: Fig. S1). The V_H_ and V_L_ sequences, joined by a peptide linker (GGGGS)_3_, were synthesized by Invitrogen GeneArt (ThermoFisher). The synthesized fragments were double-digested with NotI-HF and NcoI-HF (NEB #R3189 and #R3193) and cloned into home-made expression vectors, containing the Fc moieties of mouse (IgG2A, UniProt #P01867) or rabbit (IgG, UniProt #P01870).

For antibody production, HEK293 suspension cells (growing in FreeStyle™ 293 Expression Medium, Gibco #12338) were transiently transfected with the vector coding for the corresponding scFv-Fc. Supernatants were collected after 4 days, and antibody yields were assessed by Page Blue staining (ThermoFisher #24620) against markers of known concentrations.

#### Immunofluorescence

5 × 10^5^*D. discoideum* DH1 cells, grown axenically at 21 °C, were allowed to settle on a 22 × 22 mm glass coverslip (Menzel-Gläser) for 90 min at room temperature in HL5 medium, then fixed with HL5 + 4% (w/v) paraformaldehyde (Applichem #A3013) for 30 min, and blocked with PBS + 40 mM ammonium chloride (NH_4_Cl) (Applichem #A3661) for 5 min. Cells were then permeabilized in methanol at minus 20 °C for 2 min, washed once with PBS (5 min), and incubated for 15 min in PBS + 0.2% (w/v) BSA (PBS-BSA). Cells were then incubated for 30 min with the indicated scFv-Fc antibody. For co-labelling experiments, the original mouse hybridoma supernatant was added to this incubation, and the reformatted antibody exhibited a rabbit Fc. After 3 washes (5, 5, 15 min) with PBS-BSA, cells were incubated for 30 min with secondary goat anti-mouse IgG conjugated to AlexaFluor-488 and/or anti-rabbit IgG conjugated to AlexaFluor-647 (1:300, Molecular Probes #A11029 and #A21245). After 3 washes (5, 5, 15 min) with PBS-BSA and one wash (5 min) with PBS, coverslips were mounted on slides (Menzel-Gläser, 76 × 26 mm) with Möwiol (Hoechst) + 2.5% (w/v) DABCO (Fluka, #33480). Pictures were taken using a Zeiss LSM700 confocal microscope, with a 63 × Neofluar oil immersion objective.

### Results and discussion

#### Sequencing of monoclonal antibodies and conversion to recombinant antibodies

The production of monoclonal antibodies by hybridoma cell lines was first achieved in 1975 [8] and was a major technological development in biomedical research. Despite its enormous importance, the technique is not devoid of problems, the most serious being the possibility of losing a particular hybridoma cell line-because either the cells die or they are unable to regrow. In addition, hybridoma cell lines are genetically unstable and it is not uncommon to see a cell line lose the ability to produce antibodies [9].

One easy solution is to sequence the antibody genes directly from the hybridoma cells. This guarantees a permanent, inexpensive and flexible storage, since all the information is contained in an electronic file, and/or in a DNA plasmid [10]. Once the sequence information is available, it can easily be stored and propagated, and it can also be used to produce the corresponding recombinant antibody. A recombinant antibody has the flexibility to be produced in a variety of systems (in bacteria, fungi or mammalian cells, to give only a few examples) and formats, in particular with any protein/peptide tag or Fc region (the binding site for secondary reagents) (Additional file 1: Fig. S1). As a consequence, a mouse monoclonal antibody produced by hybridoma cells can easily be turned into a rabbit-like antibody, or decorated with affinity tags or fluorochromes (such as Myc or GFP).

In our laboratory, we have a collection of more than 70 hybridoma cell lines producing antibodies against diverse *Dictyostelium* antigens, created by us and others over the last 30 years (for a complete list, see Additional file 1: Table S2). Using well-established techniques of hybridoma sequencing, we determined the antibody sequence for 26 of them (Table 1). Twelve were converted to recombinant antibodies. For this, the two variable regions were fused to create a single-chain variable fragment (scFv), which was then joined to a mouse or rabbit Fc moiety (Table 1 and Additional file 1: Fig. S1).

**Table 1:** List of sequenced hybridomas; highlighted in bolditalic, the antibodies converted to rAbs

ABCD_^a^	Hybridoma	UniProt	DDB_	Target	Original ref.^b^	rAb ref.^b^
AN700	H9	–	–	Membrane protein p23	[11]	
***AJ155***	H194	–	–	Membrane protein p23	[11]	[12]
***AJ513***	H72	–	–	Membrane protein p25	[11]	[13]
AN701	H36	–	–	Surface protein p46	[14]	
***AJ514***	221-342-5	–	–	Common antigen 1 (CA1)	[15]	[16, 17]
AN704	173-185-1	–	–	Common antigen 1 (CA1)	[18]	
***AK426***	1/39	–	–	Golgi	[19]	[20]
***AK423***	224-236-1	P07830	several	Actin	[21]	[22, 23]
AK566	33-294-17	P08796	G0289073	Contact site A protein	[24]	
AN709	41-71-21	P08796	G0289073	Contact site A protein	[24]	
AK425	176-3-6	P27133	G0267382	Coronin A	[25]	
AN706	194-62-7	P27133	G0267382	Coronin A	[26]	
AN707	130-80-2	P21837	G0285419	Crystal protein	[27]	
AN710	80-52-13	P02886	G0273063	Discoidin 1 chain A	[28]	
***AJ154***	H161	Q7YXD4	G0287297	Endosomal membrane protein p80	[11]	[29, 30]
AN702	H191	Q7YXD4	G0287297	Endosomal membrane protein p80	[11]	
***AK421***	70-100-1	Q01501	G0271848	Porin A	[31]	[32, 33]
AK424	21-55-4	P08799	G0286355	Myosin II heavy chain	[34]	
AN708	21-96-3	P08799	G0286355	Myosin II heavy chain	[35]	
AK567	221-64-1	Q86IA3	G0276141	Protein disulfide isomerase	[36]	c
AN703	221-42-1	Q86IA3	G0276141	Protein disulfide isomerase	[36]	
***AK422***	B4.2	O77257	G0278725	Secreted protein SctA	[37]	[38]
***AJ156***	169-477-5	P0CE95	G0290481	Talin A	[39]	[40, 41]
AN705	227-341-4	P0CE95	G0290481	Talin A	[42]	
***AJ520***	221-35-2	P54647	G0287127	V-ATPase subunit A	[43]	[44, 45]
AJ515	224-256-2	P54648	G0284473	V-ATPase subunit C	[46]	c

^a^ ABCD nomenclature (https://web.expasy.org/abcd/); the ABCD database is a manually curated repository of sequenced antibodies [47]

^b^ “Original references” correspond to the first descriptions of a monoclonal antibody. “rAb references” describe the characterization of the converted rAbs

^c^ The recombinant versions of these antibodies were not produced efficiently in our hands

To characterize the converted rAbs, immunofluorescence and/or western blot experiments were performed, comparing the original IgG produced by the hybridoma cells and the derived rAb. Ten of the converted rAbs recapitulated the immunolabelling of the original antibody (Table 1 for references). In two cases (AK567 and AJ515), the converted rAbs had production yields too low to be usable and thus failed to generate a specific labeling.

**Fig. 1:**
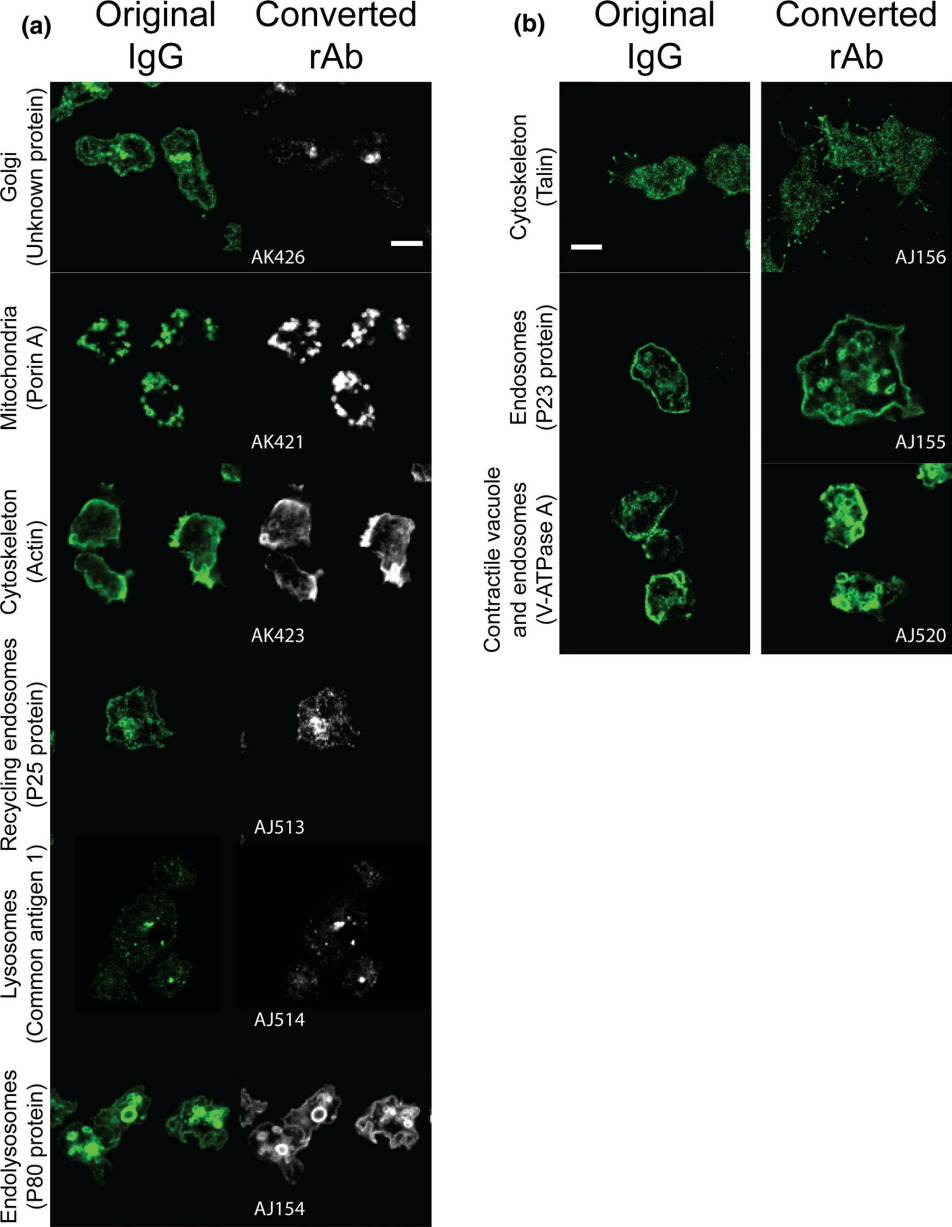
A panel of recombinant antibodies labels specific *Dictyostelium* subcellular compartments. The target antigen is indicated between parentheses. In (**a**), double immunolabelling with the original mouse IgG and the converted rAb (with a rabbit Fc) shows the same staining pattern. In (**b**), the converted rAb has a mouse Fc, and double immunolabelling could not be performed; staining patterns are identical. For more images and experimental details, please refer to the rAb references indicated in Table 1

This work yielded recombinant antibodies that can be used as markers of *Dictyostelium* subcellular compartments, notably mitochondria (AK421), Golgi apparatus (AK426), endolysosomal compartments (AJ154, AJ155, AJ513, AJ514, AJ520), cytoskeleton (AK423, AJ156), and contractile vacuole (AJ520) (Fig. 1).

#### Selection of new recombinant antibodies by phage display

Strategies to select antibodies using completely in vitro methodologies first appeared in the mid-1980s [48]. However, most academic laboratories do not have access to the phage display technology for discovery of new antibodies. Since 2013, the Geneva Antibody Facility selects, produces and characterizes recombinant antibodies for the academic community, in an open-access, not-for-profit and collaborative spirit [5].

Over the past years, we have selected 184 antibodies against 41 different *D. discoideum* proteins (Table 2) using the phage display technique. The in vitro selection has been done with a wide variety of antigen molecules, including small peptides, protein domains or fragments attached to a GST carrier, full-length proteins, and even subcellular compartments and cells. All antibodies (using the already mentioned scFv-Fc format) specifically recognize the target used for phage display discovery (a peptide, a protein fragment, a full protein) by ELISA. It should however be emphasized that an antibody directed against a peptide may fail to recognize the corresponding sequence in the full folded protein. We did not characterize in depth all the antibodies discovered; further characterization should be performed and reported by the end-user scientists themselves. We strongly encourage them to publish positive as well as negative results [5]. The recognition of an epitope by an antibody is heavily influenced by the folding state of the protein; proteins are mostly folded in ELISA or immunofluorescence experiments and largely denatured in western blot or immunohistochemistry. Consequently, antibodies that perform well with one technique often perform poorly in others [49]. It is thus of paramount importance to determine the performance of each antibody in different contexts and for different techniques.

**Table 2:** List of recombinant antibodies discovered by phage display

ABCD_	UniProt	DDB_	Target	Refs.
RB339–342	Q86B07	G0272785	Acyloxyacyl hydrolase AoaH	
RB152–154, RB186–188	Q8T7K0	G0275451	ALG-2 interacting protein X	
RB393–395	Q54Q68	G0284043	Amoebapore-like protein AplA	
RB349–357	Q54LG3	G0286651	Amoebapore-like protein AplB	
RB358–363	Q54SX7	G0282153	Amoebapore-like protein AplH	
RB345–348	Q54JE8	G0288095	Bactericidal permeability-increasing protein BpiA	
RB364–386	Q55BA2	G0271242	Bactericidal permeability-increasing protein BpiC	
RB169–178	P34090	G0275007	Conditioned medium factor CmfA	
RB155–156, RB189	Q54LJ3	G0286797	ESCRT-I complex subunit Tsg101	[55]
RB337–338	Q54DN3	G0291986	Exportin-7	
RB167–168	Q54U89	G0281211	Folate receptor Far1	[51]
RB002, RB004–005, RB009, RB045, RB048	Q54KF7	G0287363	Integrin beta-like protein SibA	[50]
RB097, RB103	Q54TM7	G0281557	Leucine-rich repeat kinase LrrkA	
RB305–306	Q9XYS8	G0267406	Lysosome membrane protein 2-A	
RB313–318	Q9BKJ9	G0287035	Lysosome membrane protein 2-B	
RB328–331	Q55FQ9	G0267440	Lysosome membrane protein 2-C	
RB343–344, RB376-378, RB464–467	Q8T1G4	G0275123	Lysozyme AlyA	[53, 56, 57]
RB388–392, RB447–453	Q54M35	G0286229	Lysozyme AlyL	[54, 58–60]
RB320–327, RB396–399	Q55GK8	G0267630	Membrane-associated sulfotransferase Kil1	
RB007, RB011–012	Q54EY0	G0291275	Mucolipin	[50]
RB285–290	Q867T7	G0288773	NADPH oxidase activator NcfA	[61]
RB436–442	Q54KA3	G0287497	Nucleoporin 133	[62, 63]
RB003, RB015–016	Q55FP0	G0267444	Phagocytic receptor 1a	[50]
RB332–336	Q54ZW0	G0277273	Phagocytic receptor 1b	
RB291–293	Q553Q2	G0275345	Rab GTPase-activating protein 1-like	
RB431–435	P18613	G0291237	Rap GTPase RapA	[64]
RB001	Q9NIV0	G0283389	Rhesus-like glycoprotein A	
RB374–375	Q54N92	G0285435	RNB domain-containing ribonuclease	
RB179–182	Q54H46	G0289791	Serine/threonine-protein kinase DrkA	
RB019–021	Q54JQ7	G0287845	Spreading and phagocytosis regulator SpdA	[52]
RB008	Q9XYS3	G0289653	Superoxide-generating NADPH oxidase heavy chain subunit A	
RB010, RB039–040, RB042	Q86GL4	G0287101	Superoxide-generating NADPH oxidase heavy chain subunit B	
RB025–026	Q54F44	G0291117	Superoxide-generating NADPH oxidase heavy chain subunit C	[50]
RB029–031, RB060–062	Q55CW7	G0269872	Tetraspanin TspB	
RB513–518	–	–	Unknown lysosomal protein	[65]
RB098–102	Q54KX3	G0287055	Vacuolar protein sorting-associated protein 13F	
RB150–151, RB183–185	Q54PT2	G0284347	Vacuolar protein sorting-associated protein 4	
RB256–267	O15706	G0289485	Vacuolin A	
RB268–269	–	–	Vacuolin ABC	
RB258–259	Q54WZ2	G0279191	Vacuolin B	
RB260–261	Q54WZ3	G0279307	Vacuolin C	

So far, antibodies to 9 targets have been described in a scientific publication, using an additional technique to ELISA, such as western blot (Table 2). Some antibodies recognize the full-length endogenous protein (SibA, Phg1a, Far1 [50, 51]), others only recognize the full-length protein when over-expressed (AlyA, AlyL, SpdA [52–54]), and others fail completely to recognize the full-length protein, overexpressed or endogenous (Tsg101 [55]).

## Conclusions

Given the relatively small size of the *Dictyostelium* scientific community, the majority of the tools and reagents are developed by the researchers themselves, and not commercially available. This is notably the case for antibodies. In addition, almost the totality of these reagents are polyclonal antibodies produced by immunizing rabbits, or monoclonal antibodies produced by mouse hybridoma cells. While polyclonal antibodies are ill-characterized reagents that should be completely phased out, monoclonal antibodies can be irretrievably lost. Since 2015, many key opinion leader scientists have published calls to employ only recombinant antibodies [9, 66].

Here, we describe the efforts of the Geneva Antibody Facility to develop recombinant antibodies against a panel of *Dictyostelium* targets, either by sequencing existing hybridoma cell lines, or by developing new antibodies using the phage display technique. We hope that these efforts will facilitate work and increase reproducibility in this scientific community. We further hope that it will encourage others to take part in this common enterprise by (i) sequencing their own hybridomas and depositing the sequences in the ABCD database (https://web.expasy.org/abcd/; [47]); (ii) selecting and characterizing antibodies to new *Dictyostelium* proteins; and (iii) publishing the results obtained with these antibodies.

## Limitations

Antibodies developed by us are not always characterized in depth, as this is out of the scope of our laboratory. We strongly urge the end-user scientists themselves to characterize and publish any positive and/or negative results, to determine the antibody efficacy in different contexts and with different techniques.

## Supplementary information

**Additional file 1: Fig S1.** Schematic representation of the conversion of an IgG into an scFv-Fc. (A) An IgG is composed of 4 chains: 2 heavy (H) and 2 light (L) chains, made of a constant (C) and a variable (V) domain. The F_V_ (variable fragment) consists of two chains (V_H_ and V_L_) and is the region responsible for antigen recognition and binding; thus, it is the region of interest for sequencing. (B) An scFv is made of the V_H_ and V_L_ variable chains joined by a peptide linker (GGGGSGGGGSGGGGS). (C) An scFv-Fc is an scFv molecule fused to an Fc region; the Fc can be of any desired species (rabbit, mouse, human, guinea pig), and it is the region where secondary reagents bind to. **Table S1.** List of degenerate primers used for hybridoma sequencing. **Table S2.** Collection of Cosson lab’s hybridoma cell lines producing antibodies against *Dictyostelium* antigens.

## Data Availability

All data generated or analyzed during this study are included in this published article and its supplementary information files. All antibodies are available at the Geneva Antibody Facility (https://www.unige.ch/medecine/antibodies/).

## Abbreviations

rAb: Recombinant antibody
scFv: Single-chain variable fragment
