# Supplementary material for: A recombinant antibody toolbox for Dictyostelium discoideum
Source: BMC Res Notes. 2020 Apr 10;13:206. doi: 10.1186/s13104-020-05048-8 (PMC7149914; doi:10.1186/s13104-020-05048-8)
Supplement: Supplementary file 1 — Additional file 1: Fig S1. Schematic representation of the conversion of an IgG into an scFv-Fc. (A) An IgG is composed of 4 chains: 2 heavy (H) and 2 light (L) chains, made of a constant (C) and a variable (V) domain. The FV (variable fragment) consists of two chains (VH and VL) and is the region responsible for antigen recognition and binding; thus, it is the region of interest for sequencing. (B) An scFv is made of the VH and VL variable chains joined by a peptide linker (GGGGSGGGGSGGGGS). (C) An scFv-Fc is an scFv molecule fused to an Fc region; the Fc can be of any desired species (rabbit, mouse, human, guinea pig), and it is the region where secondary reagents bind to. Table S1. List of degenerate primers used for hybridoma sequencing. Table S2. Collection of Cosson lab’s hybridoma cell lines producing antibodies against Dictyostelium antigens. [file 13104_2020_5048_MOESM1_ESM.docx]

Additional file 1


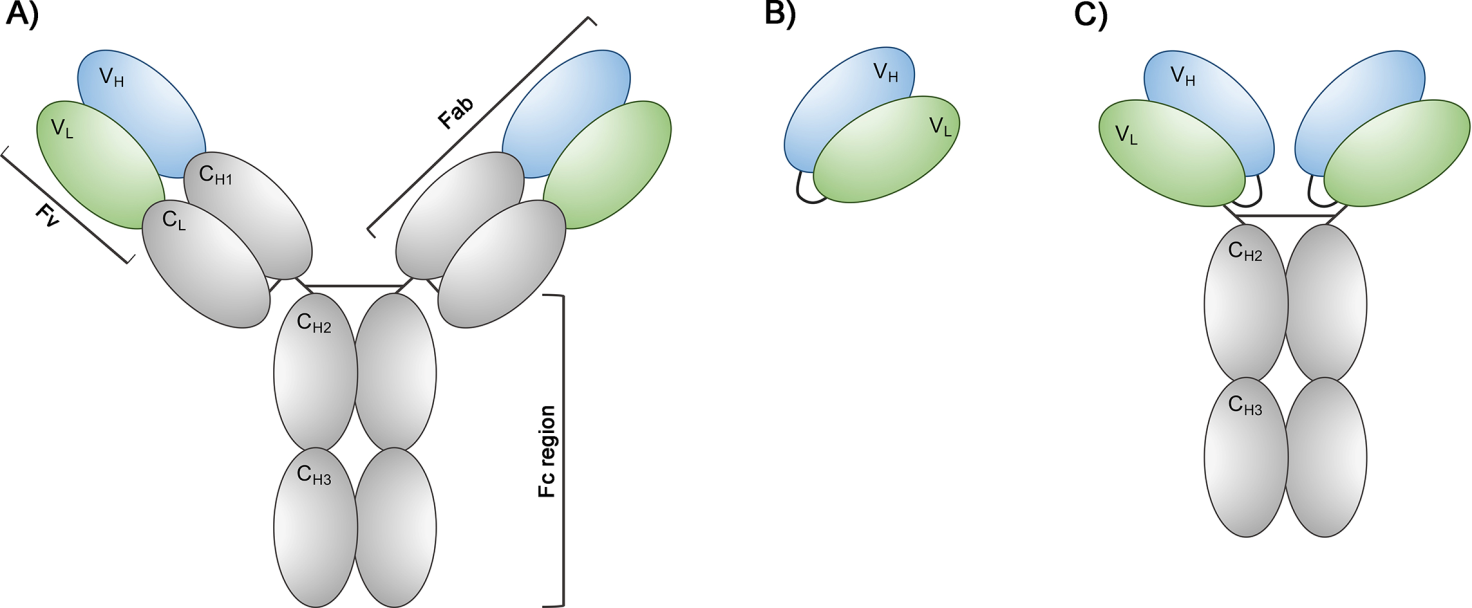


**Fig S1:** Schematic representation of the conversion of an IgG into an scFv-Fc. **(A)** An IgG is composed of 4 chains: 2 heavy (H) and 2 light (L) chains, made of a constant (C) and a variable (V) domain. The F_V_ (variable fragment) consists of two chains (V_H_ and V_L_) and is the region responsible for antigen recognition and binding; thus, it is the region of interest for sequencing. **(B)** An scFv is made of the V_H_ and V_L_ variable chains joined by a peptide linker (GGGGSGGGGSGGGGS). **(C)** An scFv-Fc is an scFv molecule fused to an Fc region; the Fc can be of any desired species (rabbit, mouse, human, guinea pig), and it is the region where secondary reagents bind to.

**Table S1**: List of degenerate primers used for hybridoma sequencing.

| **Mix** | **Oligo** | **Sequence** |
| --- | --- | --- |
| **VH- Fo** | **H-F1** | **gagaAAGCTT**GAGGTTCDSCTGCAACAGTY |
|  | **H-F2** | **gagaAAGCTT**CAGGTGCAAMTGMAGSAGTC |
|  | **H-F3** | **gagaAAGCTT**GAVGTGMWGCTGGTGGAGTC |
|  | **H-F4** | **gagaAAGCTT**CAGGTTAYTCTGAAAGAGTC |
|  | **H-F5** | **gagaAAGCTT**CAKGTGCAGCTTCAGSAGTC |
|  | **H-F6** | **gagaAAGCTT**CAGATCCAGTTSGYGCAGTC |
|  | **H-F7** | **gagaAAGCTT**CAGRTCCAACTGCAGCAGYC |
|  | **H-F8** | **gagaAAGCTT**GAGGTGMAGCTASTTGAGWC |
|  | **H-F9** | **gagaAAGCTT**GAAGTGAAGMTTGAGGAGTC |
|  | **H-F10** | **gagaAAGCTT**GATGTGAACCTGGAAGTGTC |
|  | **H-F11** | **gagaAAGCTT**CAGATKCAGCTTMAGGAGTC |
|  | **H-F12** | **gagaAAGCTT**CAGGCTTATCTGCAGCAGTC |
|  | **H-F13** | **gagaAAGCTT**CAGGTTCACCTACAACAGTC |
|  | **H-F14** | **gagaAAGCTT**CAGGTGCAGCTTGTAGAGAC |
|  | **H-F15** | **gagaAAGCTT**GARGTGMAGCTGKTGGAGAC |
| **VH- Re** | **H-R1** | **gagaCCGCGG**CGAGGAGACGGTGACMGTGG |
|  | **H-R2** | **gagaCCGCGG**CGCAGAGACAGTGACCAGAG |
|  | **H-R3** | **gagaCCGCGG**CGAGGAGACTGTGAGASTGG |
| **VLK- Fo** | **L-FK1** | **gagaAAGCTT**GACAWTGTTCTCACCCAGTC |
|  | **L-FK2** | **gagaAAGCTT**GACATCCAGATGACACAGWC |
|  | **L-FK3** | **gagaAAGCTT**GATRTTGTGATGACCCAGWC |
|  | **L-FK4** | **gagaAAGCTT**GACATTSTGMTGACCCAGTC |
|  | **L-FK5** | **gagaAAGCTT**GATGTTGTGVTGACCCAAAC |
|  | **L-FK6** | **gagaAAGCTT**GACACAACTGTGACCCAGTC |
|  | **L-FK7** | **gagaAAGCTT**GAYATTKTGCTCACTCAGTC |
|  | **L-FK8** | **gagaAAGCTT**GATATTGTGATRACCCAGGM |
|  | **L-FK9** | **gagaAAGCTT**GACATTGTAATGACCCAATC |
|  | **L-FK10** | **gagaAAGCTT**GACATTGTGATGWCACAGTC |
|  | **L-FK11** | **gagaAAGCTT**GATRTCCAGATGAMCCAGTC |
|  | **L-FK12** | **gagaAAGCTT**GATGGAGAAACAACACAGGC |
| **VLK- Re** | **L-RK1** | **gagaCCGCGG**GCGTTTBATTTCCAGCTTGG |
|  | **L-RK2** | **gagaCCGCGG**GCGTTTTATTTCCAATTTTG |
| **VLL- Re+Fo** | **L-FL1** | **gagaAAGCTT**GACGCTGTTGTGACTCAGGA |
|  | **L-FL2** | **gagaAAGCTT**GACCYTGTGCTCACTCAGTC |
|  | **L-RL** | **gagaCCGCGG**GCCTAGGACAGTCAMCYTGG |

**Table S2**: Collection of Cosson lab’s hybridoma cell lines producing antibodies against *Dictyostelium* antigens.

| **Hybridoma** | **UniProt** | **DDB_** | **Target** | **Original ref.** |
| --- | --- | --- | --- | --- |
| 224-236-1 | P07830 | several | Actin | [1] |
| 159-291-1 | P24005 | G0279081 | Actin binding protein B, ABP34 | [2] |
| 169-478-6 | P24005 | G0279081 | Actin binding protein B, ABP34 | [3] |
| 169-90-2 | P24005 | G0279081 | Actin binding protein B, ABP34 | [3, 4] |
| 188-19-91 | P13021 | G0267374 | Actin capping protein A, cap32 | [5] |
| 135-409-16 | P13022 | G0272104 | Actin capping protein B, cap34 | [6] |
| 246-153-2 | P54686 | G0278733 | Actin interacting protein 1, DAip1 | [7] |
| M12A9 | Q8I8U2 | G0281957 | AP-1 complex gamma 1 subunit | [8, 9] |
| 4/148 | Q1ZXQ8 | G0268616 | Centrosomal protein CepJ, CP224 | [10, 11] |
| 177-29-5 | P34121 | G0293898 | Coactosin, p17 | [12] |
| 190-68-1 | Q03380 | G0289599 | Comitin ComA, p24 | [13] |
| 123-353-2 | P08796 | G0289073 | Contact site A protein | [14] |
| 24-210-2 | P08796 | G0289073 | Contact site A protein | [15] |
| 33-294-17 | P08796 | G0289073 | Contact site A protein | [15] |
| 41-71-21 | P08796 | G0289073 | Contact site A protein | [15] |
| 176-3-6 | P27133 | G0267382 | Coronin A | [16] |
| 194-62-7 | P27133 | G0267382 | Coronin A | [17] |
| 130-80-2 | P21837 | G0285419 | Crystal protein | [18] |
| 80-52-13 | P02886 | G0273063 | Discoidin 1 chain A | [19] |
| H161 | Q7YXD4 | G0287297 | Endosomal membrane protein p80 | [20] |
| H191 | Q7YXD4 | G0287297 | Endosomal membrane protein p80 | (developed by P. Cosson) |
| H70 | Q7YXD4 | G0287297 | Endosomal membrane protein p80 | ** (same Ab sequence as H161)* |
| 210-183-1 | P54680 | G0277855 | Fimbrin, p67 | [21] |
| 21-55-4 | P08799 | G0286355 | Myosin II heavy chain | [22] |
| 21-96-3 | P08799 | G0286355 | Myosin II heavy chain | [23] |
| 70-100-1 | Q01501 | G0271848 | Porin A | [24] |
| 153-246-10 | P26199 | G0287125 | Profilin I | [25] |
| 221-184-2 | Q86IA3 | G0276141 | Protein disulfide isomerase | [26] |
| 221-402-9 | Q86IA3 | G0276141 | Protein disulfide isomerase | [26] |
| 221-42-1 | Q86IA3 | G0276141 | Protein disulfide isomerase | [26] |
| 221-64-1 | Q86IA3 | G0276141 | Protein disulfide isomerase | [26] |
| B4.2 | O77257 | G0278725 | Secreted protein SctA | [27] |
| 169-477-5 | P0CE95 | G0290481 | Talin A | [3] |
| 227-341-4 | P0CE95 | G0290481 | Talin A | [28] |
| 221-35-2 | P54647 | G0287127 | V-ATPase subunit A | [29] |
| 224-256-2 | P54648 | G0284473 | V-ATPase subunit C | [30] |
| 151-456-7 | P36418 | G0276453 | Villin, Cap100 | [31] |
| 173-185-1 | ---- | ---- | Common antigen 1 (CA1) | [32] |
| 221-342-5 | ---- | ---- | Common antigen 1 (CA1) | [33] |
| 1/39 | ---- | ---- | Golgi | [10] |
| 3/121 | ---- | ---- | Centrosome | [10] |
| 4/89 | ---- | ---- | Centrosome | [10] |
| H194 | ---- | ---- | Membrane protein p23 | [20] |
| H9 | ---- | ---- | Membrane protein p23 | (developed by P. Cosson) |
| H72 | ---- | ---- | Membrane protein p25 | [20] |
| H168 | ---- | ---- | Membrane protein p25 | ** (same Ab sequence as H72)* |
| H36 | ---- | ---- | Surface protein p46 | [34] |
| H4 | ---- | ---- | Unknown membrane protein | (developed by P. Cosson) |
| H37 | ---- | ---- | Unknown membrane protein | (developed by P. Cosson) |
| H47 | ---- | ---- | Unknown membrane protein | (developed by P. Cosson) |
| H48 | ---- | ---- | Unknown membrane protein | (developed by P. Cosson) |
| H49 | ---- | ---- | Unknown membrane protein | (developed by P. Cosson) |
| H50 | ---- | ---- | Unknown membrane protein | (developed by P. Cosson) |
| H60 | ---- | ---- | Unknown membrane protein | (developed by P. Cosson) |
| H67 | ---- | ---- | Unknown membrane protein | (developed by P. Cosson) |
| H68 | ---- | ---- | Unknown membrane protein | (developed by P. Cosson) |
| H73 | ---- | ---- | Unknown membrane protein | (developed by P. Cosson) |
| H75 | ---- | ---- | Unknown membrane protein | (developed by P. Cosson) |
| H79 | ---- | ---- | Unknown membrane protein | (developed by P. Cosson) |
| H81 | ---- | ---- | Unknown membrane protein | (developed by P. Cosson) |
| H96 | ---- | ---- | Unknown membrane protein | (developed by P. Cosson) |
| H99 | ---- | ---- | Unknown membrane protein | (developed by P. Cosson) |
| H156 | ---- | ---- | Unknown membrane protein | (developed by P. Cosson) |
| H162 | ---- | ---- | Unknown membrane protein | (developed by P. Cosson) |
| H165 | ---- | ---- | Unknown membrane protein | (developed by P. Cosson) |
| H181 | ---- | ---- | Unknown membrane protein | (developed by P. Cosson) |
| H196 | ---- | ---- | Unknown membrane protein | (developed by P. Cosson) |
| H206 | ---- | ---- | Unknown membrane protein | (developed by P. Cosson) |
| H230 | ---- | ---- | Unknown membrane protein | (developed by P. Cosson) |
| H240 | ---- | ---- | Unknown membrane protein | (developed by P. Cosson) |
| H245 | ---- | ---- | Unknown membrane protein | (developed by P. Cosson) |

**References**

1. Hanakam F, Albrecht R, Eckerskorn C, Matzner M, Gerisch G. Myristoylated and non-myristoylated forms of the pH sensor protein hisactophilin II: intracellular shuttling to plasma membrane and nucleus monitored in real time by a fusion with green fluorescent protein. EMBO J. 1996,15:2935-2943.

2. Weber I, Gerisch G, Heizer C, Murphy J, Badelt K, Stock A, et al. Cytokinesis mediated through the recruitment of cortexillins into the cleavage furrow. EMBO J. 1999,18:586-594.

3. Kreitmeier M, Gerisch G, Heizer C, Muller-Taubenberger A. A talin homologue of *Dictyostelium* rapidly assembles at the leading edge of cells in response to chemoattractant. J Cell Biol. 1995,129:179-188.

4. Pikzack C, Prassler J, Furukawa R, Fechheimer M, Rivero F. Role of calcium-dependent actin-bundling proteins: characterization of *Dictyostelium* mutants lacking fimbrin and the 34-kilodalton protein. Cell Motil Cytoskeleton. 2005,62:210-231.

5. Haus U, Trommler P, Fisher PR, Hartmann H, Lottspeich F, Noegel AA, et al. The heat shock cognate protein from *Dictyostelium* affects actin polymerization through interaction with the actin-binding protein cap32/34. EMBO J. 1993,12:3763-3771.

6. Hartmann H, Noegel AA, Eckerskorn C, Rapp S, Schleicher M. Ca2+-independent F-actin capping proteins. Cap 32/34, a capping protein from *Dictyostelium discoideum*, does not share sequence homologies with known actin-binding proteins. J Biol Chem. 1989,264:12639-12647.

7. Konzok A, Weber I, Simmeth E, Hacker U, Maniak M, Muller-Taubenberger A. DAip1, a *Dictyostelium* homologue of the yeast actin-interacting protein 1, is involved in endocytosis, cytokinesis, and motility. J Cell Biol. 1999,146:453-464.

8. Lefkir Y, de Chassey B, Dubois A, Bogdanovic A, Brady RJ, Destaing O, et al. The AP-1 clathrin-adaptor is required for lysosomal enzymes sorting and biogenesis of the contractile vacuole complex in *Dictyostelium* cells. Mol Biol Cell. 2003,14:1835-1851.

9. Morrissette NS, Gold ES, Guo J, Hamerman JA, Ozinsky A, Bedian V, et al. Isolation and characterization of monoclonal antibodies directed against novel components of macrophage phagosomes. J Cell Sci. 1999,112(Pt 24):4705-4713.

10. Graf R, Daunderer C, Schliwa M. Cell cycle-dependent localization of monoclonal antibodies raised against isolated *Dictyostelium* centrosomes. Biol Cell. 1999,91:471-477.

11. Graf R, Daunderer C, Schliwa M. *Dictyostelium* DdCP224 is a microtubule-associated protein and a permanent centrosomal resident involved in centrosome duplication. J Cell Sci. 2000,113(Pt 10):1747-1758.

12. de Hostos EL, Bradtke B, Lottspeich F, Gerisch G. Coactosin, a 17 kDa F-actin binding protein from *Dictyostelium discoideum*. Cell Motil Cytoskeleton. 1993,26:181-191.

13. Weiner OH, Murphy J, Griffiths G, Schleicher M, Noegel AA. The actin-binding protein comitin (p24) is a component of the Golgi apparatus. J Cell Biol. 1993,123:23-34.

14. Faix J, Gerisch G, Noegel AA. Constitutive overexpression of the contact site A glycoprotein enables growth-phase cells of *Dictyostelium discoideum* to aggregate. EMBO J. 1990,9:2709-2716.

15. Bertholdt G, Stadler J, Bozzaro S, Fichtner B, Gerisch G. Carbohydrate and other epitopes of the contact site A glycoprotein of *Dictyostelium discoideum* as characterized by monoclonal antibodies. Cell Differ. 1985,16:187-202.

16. de Hostos EL, Rehfuess C, Bradtke B, Waddell DR, Albrecht R, Murphy J, et al. *Dictyostelium* mutants lacking the cytoskeletal protein coronin are defective in cytokinesis and cell motility. J Cell Biol. 1993,120:163-173.

17. Faix J, Dittrich W, Prassler J, Westphal M, Gerisch G. pDcsA vectors for strictly regulated protein synthesis during early development of *Dictyostelium discoideum*. Plasmid. 1995,34:148-151.

18. Bomblies L, Biegelmann E, Doring V, Gerisch G, Krafft-Czepa H, Noegel AA, et al. Membrane-enclosed crystals in *Dictyostelium discoideum* cells, consisting of developmentally regulated proteins with sequence similarities to known esterases. J Cell Biol. 1990,110:669-679.

19. Wetterauer B, Jacobsen G, Morandini P, MacWilliams H. Mutants of *Dictyostelium* *discoideum* with defects in the regulation of discoidin I expression. Dev Biol. 1993,159:184-195.

20. Ravanel K, de Chassey B, Cornillon S, Benghezal M, Zulianello L, Gebbie L, et al. Membrane sorting in the endocytic and phagocytic pathway of *Dictyostelium discoideum*. Eur J Cell Biol. 2001,80:754-764.

21. Prassler J, Stocker S, Marriott G, Heidecker M, Kellermann J, Gerisch G. Interaction of a *Dictyostelium* member of the plastin/fimbrin family with actin filaments and actin-myosin complexes. Mol Biol Cell. 1997,8:83-95.

22. Pagh K, Gerisch G. Monoclonal antibodies binding to the tail of *Dictyostelium discoideum* myosin: their effects on antiparallel and parallel assembly and actin-activated ATPase activity. J Cell Biol. 1986,103:1527-1538.

23. Claviez M, Pagh K, Maruta H, Baltes W, Fisher P, Gerisch G. Electron microscopic mapping of monoclonal antibodies on the tail region of *Dictyostelium* myosin. EMBO J. 1982,1:1017-1022.

24. Troll H, Malchow D, Muller-Taubenberger A, Humbel B, Lottspeich F, Ecke M, et al. Purification, functional characterization, and cDNA sequencing of mitochondrial porin from *Dictyostelium discoideum*. J Biol Chem. 1992,267:21072-21079.

25. Haugwitz M, Noegel AA, Rieger D, Lottspeich F, Schleicher M. *Dictyostelium discoideum* contains two profilin isoforms that differ in structure and function. J Cell Sci. 1991,100(Pt 3):481-489.

26. Monnat J, Hacker U, Geissler H, Rauchenberger R, Neuhaus EM, Maniak M, et al. *Dictyostelium discoideum* protein disulfide isomerase, an endoplasmic reticulum resident enzyme lacking a KDEL-type retrieval signal. FEBS Lett. 1997,418:357-362.

27. Sabra A, Leiba J, Mas L, Louwagie M, Coute Y, Journet A, et al. Pycnosomes: condensed endosomal structures secreted by *Dictyostelium* amoebae. PLoS One. 2016,11:e0154875.

28. Niewohner J, Weber I, Maniak M, Muller-Taubenberger A, Gerisch G. Talin-null cells of *Dictyostelium* are strongly defective in adhesion to particle and substrate surfaces and slightly impaired in cytokinesis. J Cell Biol. 1997,138:349-361.

29. Neuhaus EM, Horstmann H, Almers W, Maniak M, Soldati T. Ethane-freezing/methanol-fixation of cell monolayers: a procedure for improved preservation of structure and antigenicity for light and electron microscopies. J Struct Biol. 1998,121:326-342.

30. Journet A, Chapel A, Jehan S, Adessi C, Freeze H, Klein G, et al. Characterization of *Dictyostelium discoideum* cathepsin D. J Cell Sci. 1999,112(Pt 21):3833-3843.

31. Hofmann A, Eichinger L, Andre E, Rieger D, Schleicher M. Cap100, a novel phosphatidylinositol 4,5-bisphosphate-regulated protein that caps actin filaments but does not nucleate actin assembly. Cell Motil Cytoskeleton. 1992,23:133-144.

32. Schopohl D, Muller-Taubenberger A, Orthen B, Hess H, Reutter W. Purification and properties of a secreted and developmentally regulated alpha-L-fucosidase from *Dictyostelium discoideum*. J Biol Chem. 1992,267:2400-2405.

33. Knecht DA, Dimond RL, Wheeler S, Loomis WF. Antigenic determinants shared by lysosomal proteins of *Dictyostelium discoideum*. Characterization using monoclonal antibodies and isolation of mutations affecting the determinant. J Biol Chem. 1984,259:10633-10640.

34. Mercanti V, Charette SJ, Bennett N, Ryckewaert JJ, Letourneur F, Cosson P. Selective membrane exclusion in phagocytic and macropinocytic cups. J Cell Sci. 2006,119:4079-4087.
